# Nonadaptive female pursuit of extrapair copulations can evolve through hitchhiking

**DOI:** 10.1002/ece3.3915

**Published:** 2018-03-06

**Authors:** Nan Lyu, Maria R. Servedio, Yue‐Hua Sun

**Affiliations:** ^1^ Key Laboratory of Animal Ecology and Conservation Biology Institute of Zoology Chinese Academy of Sciences Beijing China; ^2^ Department of Biology University of North Carolina Chapel Hill NC USA; ^3^ Ministry of Education Key Laboratory for Biodiversity and Ecological Engineering College of Life Sciences Beijing Normal University Beijing China

**Keywords:** extrapair copulation, female pursuit behavior, linkage disequilibrium, nonadaptive model, sexually antagonistic coevolution

## Abstract

Mounting evidence has indicated that engaging in extrapair copulations (EPCs) might be maladaptive or detrimental to females. It is unclear why such nonadaptive female behavior evolves. In this study, we test two hypotheses about the evolution of female EPC behavior using population genetic models. First, we find that both male preference for allocating extra effort to seek EPCs and female pursuit behavior without costs can be maintained and remain polymorphic in a population via frequency‐dependent selection. However, both behaviors cannot evolve when females with pursuit behavior suffer from a decline in male parental care. Second, we present another novel way in which female pursuit behavior can evolve; indirect selection can act on this behavior through a ratchet‐like mechanism involving oscillating linkage disequilibria between the target EPC pursuit locus and two other loci determining male mate choice and a female sexual signal. Although the overall positive force of such indirect selection is relatively weak, our results suggest that it may still play a role in promoting the evolution of female EPC behavior when this behavior is nonadaptive (i.e., it is neutral) or only somewhat maladaptive (e.g., males only occasionally lower parental care when their mates pursue EPCs).

## 1 INTRODUCTION

In many socially monogamous species, individuals have been found to engage in extrapair copulations (EPC), which results in extrapair paternity (EPP) (Westneat & Stewart, 2003). EPP has been recorded in about 90% of investigated avian species (reviewed in Griffith, Owens, and Thuman (2002)) and also in other animal taxon, such as mammals (e.g., Cohas & Allainé, 2009; Goossens et al., 1998; Palombit, 1994; Reichard, 1995). Although extrapair matings certainly have the potential to increase the offspring production of males (Trivers, 1972), the evolution of such behavior in females is less clear (Forstmeier, Martin, Bolund, Schielzeth, & Kempenaers, 2011). The reason why females accept or even actively solicit or pursue EPCs (e.g., Cockburn et al., 2009; Forstmeier, 2007; Sheldon, 1994) is a long‐standing problem that has puzzled biologists for over two decades (Arnqvist & Kirkpatrick, 2005; Eliassen & Kokko, 2008; Forstmeier, Nakagawa, Griffith, & Kempenaers, 2014) and still stimulates strong interest (Arct, Drobniak, & Cichoń, 2015).

Both adaptive and nonadaptive hypotheses have been proposed to explain female pursuit behavior (Forstmeier et al., 2014). Adaptive hypotheses state that females should benefit from such behavior, by gaining higher offspring heterozygosity (Mays, Albrecht, Liu, & Hill, 2008), good genes through mating with higher‐quality extrapair males (Kempenaers et al., 1992) or fertility assurance (Wetton & Parkin, 1991). However, the empirical evidence for these adaptive hypotheses has been controversial (Arnqvist & Kirkpatrick, 2005; Forstmeier et al., 2014). Nonadaptive hypotheses state that female EPC behavior can evolve and be maintained as a by‐product of selection on other traits even if there is no direct benefit to females (Forstmeier et al., 2014; Halliday & Arnold, 1987; Hsu, Schroeder, Winney, Burke, & Nakagawa, 2015). Nonadaptive explanations had long been neglected, until the first empirical test by Forstmeier et al. (2011). They found a significant genetic correlation between the male and female EPC behavior and suggested that female EPC behavior might evolve through pleiotropy or linkage disequilibrium with male EPC behavior. Nonetheless, they did not pursue these suggestions empirically. It still remains unclear whether nonadaptive female EPC behavior can evolve as suggested in Forstmeier et al. (2011), or in another nonadaptive way. Therefore, further studies of the evolutionary mechanisms for nonadaptive female pursuit behavior are necessary, especially using a theoretical approach to test the feasibility of proposed hypotheses. In this study, we would like to examine conditions where EPCs evolve due to indirect selection rather than direct selection.

In many animals, males can provide significant contributions by caring for offspring with their social mate, but they also seek EPCs. The trade‐off between parental care and seeking additional matings by males can have potentially important and often overlooked influences on the evolution of parental effects and on postmating and cryptic choice (Lyu, Servedio, Lloyd, & Sun, 2017; Magrath & Komdeur, 2003; Sheldon, 2000). In this paper, we will explore two nonadaptive hypotheses concerning the effects of this trade‐off by males on the evolution of EPC behavior in females. We first ask whether different allocation behaviors by males (i.e., providing better parental care or alternatively allocating more effort to seek EPCs) can influence the evolution of nonadaptive female pursuit behavior indirectly. Here, we consider the EPC behaviors of the two sexes to be determined by two different loci. We find that different preferences for EPCs within each sex may result in unequal extrapair mating, producing linkage disequilibrium between the two loci. We demonstrate that female pursuit behavior may evolve and be maintained through this linkage disequilibrium when male preference for EPCs is positively selected and hypothesize that this may account for some cases of female EPC seeking behavior in the absence of costs, as suggested by Forstmeier et al. (2011). We refer to this hypothesis as a “two‐locus hitchhiking” model.

A second hypothesis that may account for the evolution of EPC behavior in females comes from sexually antagonistic coevolution (SAC, Arnqvist and Rowe (2005)). Here, we consider how key genetic covariances that underlie indirect selection on female EPC behavior might arise from evolving mate choice behavior during SAC. It has been demonstrated that interlocus SAC may create long‐term indirect selection that increases the frequency of alleles at other loci (e.g., a modifier of recombination rate has been shown to evolve in this manner in Dapper and Lively (2014)). We hypothesize that this mechanism may also play an important role in promoting the evolution of female EPC behavior. Specifically, it was previously shown that male postpairing mate choice and female signaling may become sexual antagonistic traits (i.e., each sex can promote the spread of a genotype that is ultimately maladaptive in the other sex) under certain ranges of cost and benefit ratios, when no particular genotype is universally better at manipulating or resisting the other genotypes (Lyu et al., 2017). We expect that such interlocus SAC of male choice and female signaling may have the potential to cause the cycling of linkage disequilibrium, which may create indirect selection on a third locus representing the female EPC behavior (as with the mechanism seen in Dapper and Lively (2014)). We refer to this hypothesis as a “three‐locus male choice” model and demonstrate its feasibility below.

## 2 CAN FEMALE EPC BEHAVIOR EVOLVE BY HITCHHIKING WITH MALE PREFERENCE FOR EPC?

### 2.1 Basic assumptions of the “two‐locus hitchhiking” model

We consider haploid genetics for the sake of simplicity. The first locus (P), expressed only in males, determines the effort that males allocate between seeking EPCs and providing parental care. The second locus (E), only expressed in females, controls whether individuals are willing to accept or even pursue EPCs. This yields four genotypes of P_1_E_1_, P_1_E_2_, P_2_E_1_, and P_2_E_2_. We assume that P_2_ males will always allocate more effort to seek EPCs and can gain benefits from extrapair fertility, but at the same time their behavior depresses their within‐pair fitness due to their reduced amount of paternal care relative to P_1_ males. Specifically, we assume that P_1_ males will allocate certain effort (*c*) to seek EPCs and unit investment (1) on parental care, while P_2_ males will allocate additional *d*_c_ effort to seek EPCs by reducing the parental care investment to 1 − δ.

For females, we assume that E_2_ females will always accept or even pursue EPCs, resulting in a proportion τ of offspring that are extrapair. Although E_1_ females are more resistant to EPCs than E_2_ ones, they may still be involved in EPCs (Arnqvist & Kirkpatrick, 2005). We therefore assume that each E_1_ female will accept EPCs and allow fertilization with a probability μ, and thus, a proportion μτ of their offspring will be extrapair. Note that these assumptions imply that the proportion of extrapair matings is controlled solely by the female genotype, regardless of whether males are P_1_ or P_2_. This is supported by evidence that the fertilization patterns are controlled by the female to a large extent (Lifjeld & Robertson, 1992). They also imply that male effort in seeking EPCs affects the success of P_2_ versus P_1_ males in obtaining extrapair matings (e.g., via competition), but does not increase a male's success with females beyond this competitive effect.

Additionally, we assume that E_2_ females may pay a cost due to their pursuit behavior. The most significant and general cost for those females is thought to be the retaliatory withholding of paternal care by their social mates (Clutton‐Brock, 1991; Trivers, 1972). Therefore, we assume that E_2_ females will suffer from a decline in male parental care by δ′. This means that the male parental care would become 1 − δ′ and 1 – δ − δ′ for P_1_ and P_2_ males, respectively, when they mate with E_2_ females. Further assumptions include: the population is discrete‐generation with a monogamous social mating system and random mating; all males and females mate and have equal within‐pair mating rates; there is no variability of quality of parents or offspring; and males provide only sperm but no parental care to their EPC mates, which should be a prevalent situation in nature (Jennions & Petrie, 2000).

### 2.2 “Two‐locus hitchhiking model” construction

We first denote the frequencies of four genotypes listed above as *x*_1_, *x*_2_, x_3_ and x_4_, respectively (Table S1). The detailed life cycle is as follows. First, males and females pair randomly. The number of surviving offspring ($\emptyset_{\mathrm{ij}}$) produced by a *x*_*i*_ female mating with a *x*_*j*_ male is assumed to be directly determined by the parental care investment (Ihara, 2002; see Table S1):

(1) $$\emptyset_{\mathrm{ij}}=1+bm_{\mathrm{ij}},$$

The first term (i.e., 1) and the second term (i.e., *bm*_*ij*_) in Equation (1) represent the surviving offspring due to the female's and male's care, respectively, where *b* represents the translation of male parental care converted to surviving offspring relative to the female's care, and *m*_*ij*_ is the expected male parental care effort. According to our above assumptions, we have *m*_*ij*_ = 1 − *d*_1_δ − *d*_2_δ′, where *d*_1_ = 1 if *j* = 3 or 4 (i.e., males have the P_2_ allele) and *d*_1_ = 0 otherwise, *d*_2_ = 1 if *i* is even (i.e., females have the E_2_ allele) and *d*_2_ = 0 otherwise.

Among those offspring produced by *x*_*i*_ females, a proportion of

(2) $$\theta_{i}=\left(1-f+f\mu \right)\tau$$

are sired by extrapair fathers, where *f* = 0 if *i* is even (i.e., females have the E_2_ allele), and *f* = 1 otherwise. The total available extrapair fertilities in the population are therefore equal to $\sum_{\mathrm{ij}}x_{i}x_{j}\emptyset_{\mathrm{ij}}\theta_{i}$. We assume that the population is large, and males perform EPC matings randomly among the whole population; the extrapair fecundity benefits gained from EPCs by *x*_*j*_ males should be linearly related to their allocated effort, *C*_*j*_ = *c* + *h*_1_ *d*_c_, where *h*_1_ = 1 if *j* = 3 or 4 (i.e., males have the P_2_ allele), and *h*_1_ = 0 otherwise. We can therefore obtain the proportion of extrapair offspring sired by *x*_*j*_ males in the population as

(3) $$\rho_{x_{j}}=\frac{x_{j}C_{j}}{\sum_{j}x_{j}C_{j}}.$$

This model results in a 4 × 4 matrix **F** of the proportion of surviving offspring between each genotype, where *F*_*ij*_ includes both within‐pair ($F_{\mathrm{ij}}^{\mathrm{in}}$) and extrapair offspring ($F_{\mathrm{ij}}^{\mathrm{ex}}$), that is, $F_{\mathrm{ij}}=F_{\mathrm{ij}}^{\mathrm{in}}+F_{\mathrm{ij}}^{\mathrm{ex}}$, where

(4) $$\begin{array}{cc} & F_{\mathrm{ij}}^{\mathrm{in}}=\frac{x_{i}x_{j}\emptyset_{\mathrm{ij}}\left(1-\theta_{i}\right)}{w},\;\;\;\text{and}\\ & F_{\mathrm{ij}}^{\mathrm{ex}}=\frac{\rho_{x_{j}}\sum_{j}x_{i}x_{j}\emptyset_{\mathrm{ij}}\theta_{i}}{w}, \end{array}$$

where $w=\sum_{\mathrm{ij}}x_{i}x_{j}\emptyset_{\mathrm{ij}}$ represents the total surviving offspring in the population.

Following the standard equations for recombination and segregation for two loci in haploids, we can derive the recursion equations for the genotype frequencies and then convert these into equations for the allele frequencies and linkage disequilibrium, *D*. Subsequently, we can deduce the equilibria of the model and calculate the local stability of each equilibrium using a linear stability analysis. Details of the analyses are archived in *Mathematica* files on Dryad (to be submitted upon acceptance).

### 2.3 Results—two‐locus hitchhiking model

First, if the female pursuit behavior (i.e., allele E_2_) is nonadaptive, but not costly or maladaptive (i.e., δ′ = 0), we can deduce from the recursion equations that the frequency of allele E_2_ changes only when the frequency of P_2_ changes (i.e. $\Delta e_{2}=\Delta p_{2}\left(D/\left(p_{2}\left(1-p_{2}\right)\right)\right)$, see Appendix S1). Therefore, the female pursuit behavior will always be in equilibrium whenever the male's allocating behavior is in equilibrium. It also implies that the selection operates on the allele E_2_ indirectly through the linkage disequilibrium between the two loci, which is the same situation as in Kirkpatrick's classic model exploring the evolution of male secondary sexual characteristic through female choice (Kirkpatrick, 1982).

**Figure 1:**
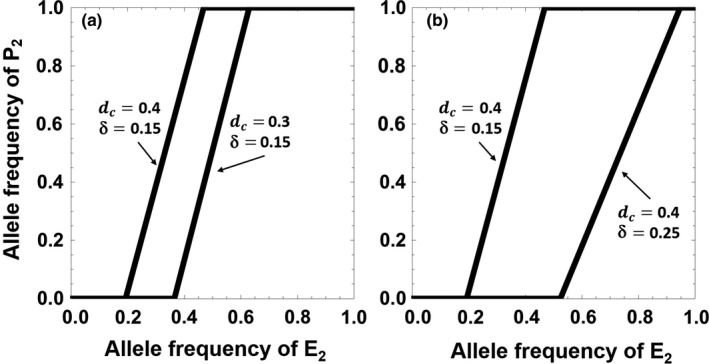
The neutrally stable equilibria of the model when (a) the extra EPC effort *d*_c_ decreases or (b) the reduction in parental care investment δ increases. The parameter values are: μ = 0.3, τ = 0.3, *b* = 0.8, *c* = 0.9. The parameter values of *d*_c_ and δ of each line are indicated directly in the figures

Specifically, we find that each of the points on the edges of *p*_2_ = 0 and *p*_2_ = 1 and on the line of

(5) $$p_{2}\;=\;\left(\frac{1+b}{b\delta }+\frac{c}{d_{c}}\right)\left(\tau -\mu \tau \right)e_{2}-\frac{c}{d_{c}}\left(1-\mu \tau \right)+\frac{1+b}{b\delta }\mu \tau$$

is an equilibrium. Stability analyses reveal that the equilibrium points on the edge of p_2_ = 0 are neutrally stable when $e_{2}<\frac{d_{c}\mu \tau \;+\;b\left(c\delta \left(1\;-\;\mu \tau \right)\;-\;d_{c}\mu \tau \right)}{\left(d_{c}\;+\;bd_{c}\;+\;bc\delta \right)\left(1\;-\;\mu \right)\tau }$ and are unstable otherwise. The equilibrium points on the edge of *p*_2_ = 1 require $e_{2}>\frac{d_{c}\mu \tau \;+\;b\left(d_{c}\delta \;-\;d_{c}\mu \tau \;+\;c\delta \left(1\;-\;\mu \tau \right)\right)}{\left(d_{c}+bd_{c}\;+\;bc\delta \right)\left(1\;-\;\mu \right)\tau }$ to be neutrally stable. Moreover, numerical analyses indicate that those equilibrium points on the line given by the Equation (1) are also neutrally stable. Therefore, the model has a set of stable equilibria that form the heavy line in Figure 1. We can see that when the extra EPC effort d_c_ decreases or the reduction in the parental care investment δ increases, this line of equilibria will shift significantly toward higher frequencies of the female pursuit allele E_2_ and lower frequencies of the male EPC allele P_2_ (Figure 1), making female EPC pursuit behavior evolve over a smaller range of starting frequencies.

This set of equilibria results in part from a trade‐off between providing parental care and seeking EPCs by males, explaining the existence of polymorphic equilibria at the P locus. Furthermore, we find that the linkage disequilibrium between the loci P and E at the line of polymorphic equilibria is always positive. The alleles P_2_ and E_2_ (and P_1_ and E_1_) are therefore statistically associated along this line. Recall that E_2_ females produce more extrapair offspring than E_1_ females and that P_2_ males allocate more efforts to seek EPCs than P_1_ males in the population. This enables P_2_ males to have a higher probability of producing extrapair offspring with E_2_ females than do P_1_ males, which results in the positive linkage disequilibrium observed. The position of the line of polymorphic equilibria thus results from the joint action of trade‐offs at the P locus and the linkage disequilibrium between P and E.

Second, what if the female pursuit behavior is costly due to a punitive decline in male parental care (i.e., δ′ > 0)? The E_2_ allele, in this case, will not be able to evolve (this again parallels the effect of a cost on preference in Kirkpatrick's sexual selection model; see Bulmer, 1989). We calculate the equilibria in this situation and find that the four points at which variation is lost at the P and E loci, and two points on the edges of this space, are equilibria (see Appendix S1). Furthermore, the stable equilibria always entail the loss of the E_2_ allele (see Appendix S1), and therefore, costly female pursuit behavior cannot evolve through the selection pressure of increasing male EPC behavior.

In summary, although female pursuit behavior can evolve when it is not costly, those equilibrium points are neutrally stable and cannot be attained when potential costs arise. Other mechanisms are therefore required to overcome such costs. Additionally, even if there is no cost (and no additional benefit, e.g., good genes) for females with pursuit behavior, the initial emergence or increase of this allele beyond low frequencies still requires an explanation (Figure 1).

## 3 CAN FEMALE EPC BEHAVIOR EVOLVE INDIRECTLY THROUGH INTERLOCUS SAC?

### 3.1 Basic assumptions of the “three‐locus male choice” model

We add into the above two‐locus model a third locus S, which determines the expression of sexual signaling traits in females that are preferred by selective males (Lyu et al., 2017). Specifically, we assume that P_2_ males will allocate more effort (*c* + *d*_c_) to seek EPCs when they mate with nonpreferred S_1_ females (i.e., without the signaling traits), but will lower their effort by *c* − *d*_c_ when they mate with preferred S_2_ females. Simultaneously, their parental care effort will become 1 − δ and 1 + δ, respectively, as a trade‐off. Furthermore, we assume that expressing the trait is costly for S_2_ females, in that such females suffer a direct mortality cost (*t*) after mating, such as occurs after the production of blue eggs in birds (Siefferman, Navara, & Hill, 2006). Normally, males would desert their current clutches if their female mate dies, possibly because they are physically incapable of incubation (Duckworth, 1992). This means that an S_2_ female can only raise her offspring successfully at a probability of 1 − *t* relative to that for an S_1_ female.

As has been previously demonstrated (Lyu et al., 2017), the two autosomal loci of P and S represent sexual antagonistic traits when P_2_ males have relatively intense changes in parental care (i.e., high value of δ) and EPC effort (i.e., high value of *d*_c_). In this situation, the allele frequencies of P_2_ and S_2_ and the linkage disequilibrium between them exhibit cyclical behavior (Lyu et al., 2017). A thorough exploration of the model with two loci (i.e., the locus P and the locus S) can be found in Lyu et al. (2017). As in the “two‐locus hitchhiking” model presented above, the third locus E is assumed to determine female EPC behavior. Note that this model reduces to the two‐locus model above when all females do not express the sexual signals, that is, when the allele frequency of S_2_ is equal to zero.

### 3.2 “Three‐locus male choice” model construction

We assume that the three loci are located on a chromosome with free recombination. These three loci therefore lead to the eight genotypes P_1_S_1_E_1_, P_1_S_1_E_2_, P_1_S_2_E_1_, P_1_S_2_E_2_, P_2_S_1_E_1_, P_2_S_1_E_2_, P_2_S_2_E_1_, and P_2_S_2_E_2_, with frequencies of *x*_1_ through *x*_8_. In comparison with the Equation (1) in the above two‐locus model, the matrix of surviving offspring for each *x*_*i*_ female mated with a *x*_*j*_ male in this three‐locus model is

(6) $$\emptyset_{\mathrm{ij}}=[1\;+\;b\left(1\;+\;k_{1}\delta \;+\;k_{2}\delta^{\prime }\right)]\left(1\;-\;k_{3}t\right),$$

where *k*_1_ = −1 if *j* > 4 (i.e., males have the P_2_ allele) and *i* = 1, 2, 5, or 6 (i.e., females have the S_1_ allele), *k*_1_ = 1 if *j* > 4 and *i* = 3, 4, 7, or 8 (i.e., females have the S_2_ allele) and *k*_1_ = 0 otherwise, *k*_2_ = −1 if *i* is even (i.e., females have the E_2_ allele) and *k*_2_ = 0 otherwise, *k*_3_ = 1 if *i* = 3, 4, 7, or 8 (i.e., females have the S_2_ allele) and *k*_3_ = 0 otherwise (see Table S2).

Note that the Equation (2) (i.e., proportion of the extrapair offspring produced by *x*_*i*_ females, θ_*i*_) is still valid for this three‐locus model. The effort allocated to seek EPCs (*C*_*ij*_) by *x*_*j*_ males mating with *x*_*i*_ females in this model can be rewritten as *C*_*ij*_ = *c* + *h*_2_ *d*_c_, where *h*_2_ = 1 if *j* > 4 (i.e., males have the P_2_ allele) and *i* = 1, 2, 5, or 6 (i.e., females have the S_1_ allele), *h*_2_ = −1 if *j* > 4 and *i* = 3, 4, 7, or 8 (i.e., females have the S_2_ allele), and *h*_2_ = 0 otherwise. Then, we obtain the proportion of extrapair offspring sired by *x*_*j*_ males in the population as

(7) $$\rho_{x_{j}}=\frac{x_{j}\sum_{i}x_{i}C_{\mathrm{ij}}}{\sum_{\mathrm{ij}}x_{i}x_{j}C_{\mathrm{ij}}}.$$

Using Equations (2), (4), (6), and (7), we derive the matrix **F** of the proportion of surviving offspring between each genotype combination in this model. The recursion equations can be derived for each genotype after assuming that recombination and segregation occur following the standard equations for three loci in haploids. We obtain the frequencies of eight genotypes in the next generation with cumbersome expressions that are not shown here, but which can be found in the *Mathematica* files on Dryad (to be submitted upon acceptance).

### 3.3 Results—”three‐locus male choice” model

**Figure 2:**
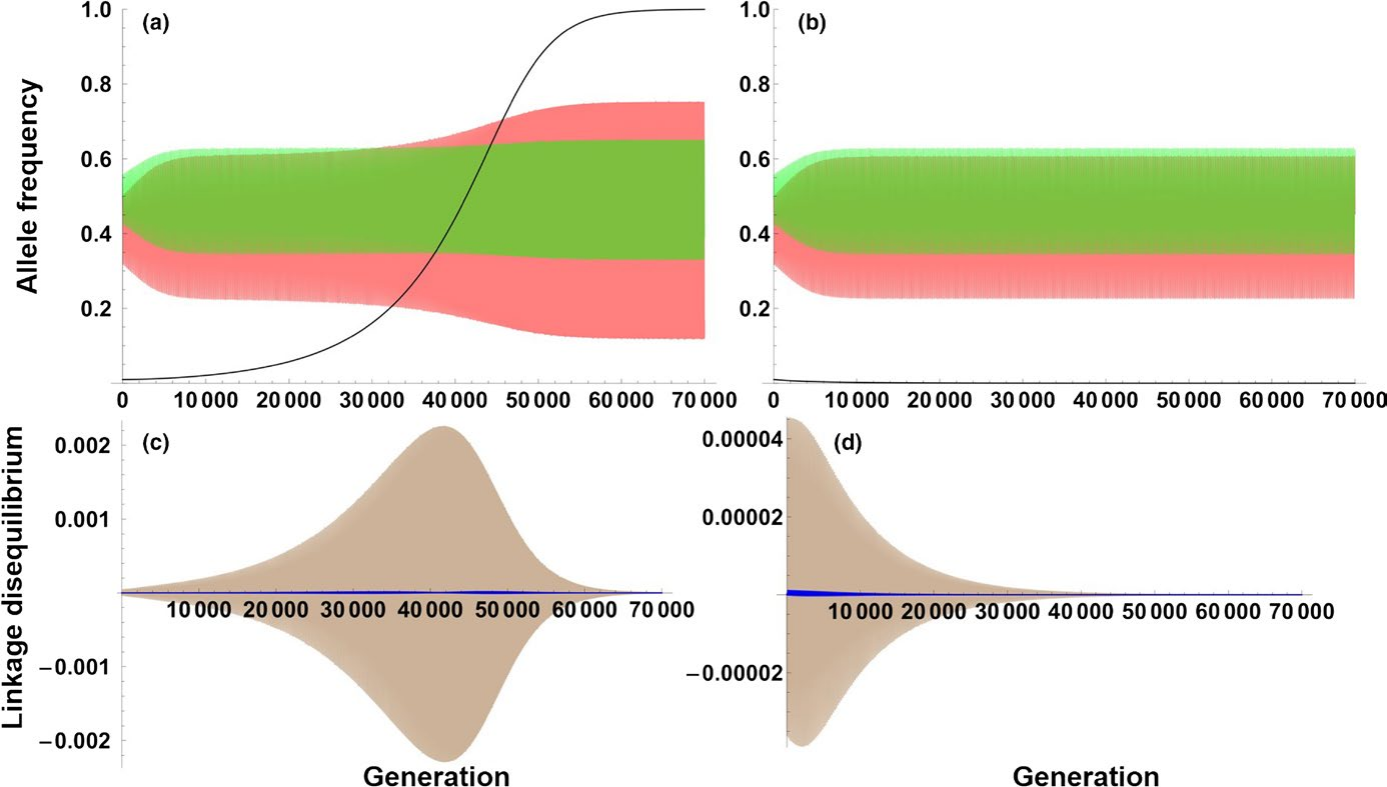
The dynamics of allele frequencies of p_2_ (red curve), s_2_ (green curve), and e_2_ (black curve) in (a) and (b) and linkage disequilibrium of *D*_PE_ (brown curve) and *D*_SE_ (blue curve) in (c) and (d). (a) and (c) δ′ = 0.00001, (b) and (d) δ′ = 0.001. E_2_ does not increase in (b) and (d). These curves consist of rapid oscillations as shown in Figures S3 and S4. The other parameter values are: *b* = 0.8; *c* = 0.8; *t* = 0.07; δ = 0.2; μ = 0.5; τ = 0.5; *d*_c_ = 0.78

**Figure 3:**
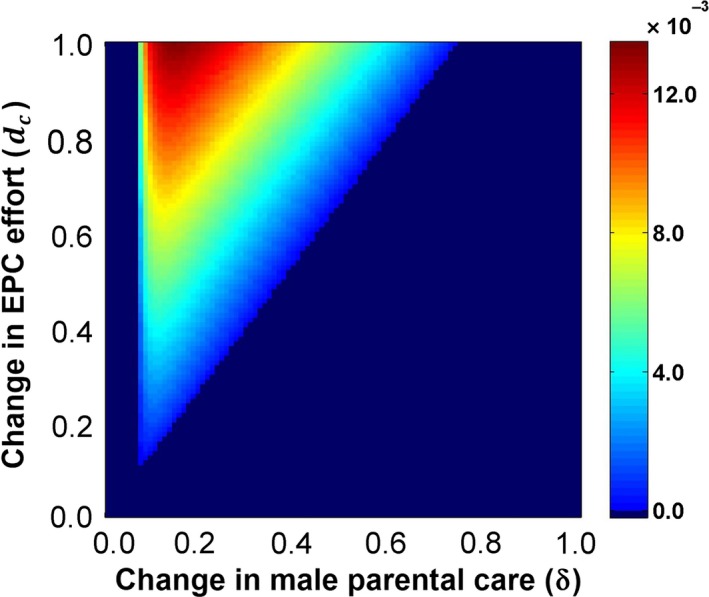
The net change in the frequency of the female pursuit allele (E_2_) after 5,000 generations under different possible combinations of the change in parental care (δ) and the change in EPC effort (*d*_c_) (ranging from 0 to 1 at a step of 0.01) when cycling occurs. Note that within those dark blue areas, either the locus P or the locus S loses variation and achieves linkage equilibrium after a number of generations, causing the allele E_2_ to stop evolving (we set the net change values within these blue areas, which are quite low, to zero for illustration). The other parameter values are: *b* = 0.7; *c* = 1.0; *t* = 0.05; δ′ = 0.0; μ = 0.5; and τ = 0.5

Here, we are primarily concerned with whether the allele E_2_ representing female pursuit behavior can evolve through indirect selection resulting from the interlocus SAC between the male choice behavior and female signaling traits. Therefore, we conduct numerical iterations of the recursion equations with the starting frequency of the allele E_2_ at 0.01 to explore the fate of a new mutation for a female with pursuit behavior that enters the population. These simulations reveal that the E_2_ allele can evolve and be maintained under conditions in which sexual antagonistic selection leads to the buildup of oscillating linkage disequilibria between the locus P and the locus E and between the locus S and the locus E (Figure 2). Numerical analyses show that the allele E_2_ can evolve especially under moderate changes in parental care (i.e., δ) and EPC effort (i.e., *d*_c_) (Figure 3). If δ is very small or is large, and/or *d*_c_ is quite small, variation will be lost at the P and S loci, that is, achieving linkage equilibrium. The allele E_2_ cannot evolve in these situations (dark blue area in Figure 3). Additionally, note that the indirect selection from linkage disequilibrium is quite weak in this model, and if the positive indirect selection cannot outperform the direct negative selection pressure from stronger costs to female pursuit behavior (Figures S2c,d and S5), the frequency of the E_2_ allele will drop down to zero, even when the locus P and the locus S are cycling (Figure 2b).

In our previous study, we assumed asymmetric values of the changes in parental care and extrapair effort by P_2_ males mating with S_1_ and S_2_ females (Lyu et al., 2017). We also ran the three‐locus model numerically under asymmetric scenarios in this study and found the same phenomenon as under the symmetric scenarios (see Figures 2 vs. S1). Therefore, we will focus on the symmetric scenario for the following analytical and numerical analyses to unravel the evolutionary mechanisms at play.

To further examine the effect of SAC at the P and S loci on the female pursuit allele E_2_, we derived an expression of the recursion equation for *e*_2_ using the notation of Barton and Turelli (1991) (see Appendix S2). This notation allows the extraction of the specific strengths of direct and indirect selection on the allele E_2_ $\left(\Delta e_{2}=\Delta e_{2}^{\mathrm{dir}}+\Delta e_{2}^{\mathrm{ind}}\right)$ (Barton & Servedio, 2015). We find that the frequency dynamics of the allele E_2_ are determined by the negative direct selection resulting from the decrease in male parental care (i.e., δ′) when females engage in more EPCs ($\Delta e_{2}^{\mathrm{dir}}=-(\left(e_{1}e_{2}b\delta^{\prime }\right)/2w)\left(1-s_{2}t\right)$) and the indirect selection brought to the E locus by linkage disequilibria with the P and S loci ($\Delta e_{2}^{\mathrm{ind}}=a_{P}D_{\mathrm{PE}}+a_{s}D_{\mathrm{SE}}$ ) (see Appendix S2 for expressions for *a*_p_ and *a*_s_, which represent direct selection on the P and S loci, respectively).

Through mapping the dynamics of the above two selection expressions (i.e., $\Delta e_{2}^{\mathrm{dir}}$ and $\Delta e_{2}^{\mathrm{ind}}$), we find that if there is no cost for the females with pursuit behavior, or if the cost is quite small, there will be stronger and longer‐term positive indirect selection (applied by the cycling behavior of P and S‐ see Appendix S3) than negative direct selection on the E_2_ allele (Figures S2a,b and S5a,c,e), thereby enabling the evolution of female pursuit behavior until E_2_ reaches fixation (Figure 2a). The fact that linkage disequilibrium between the loci P and E switches sign from positive to negative roughly at the same time as the selection favoring P_2_ switches sign from positive to negative explains why the term remains positive, causing a ratcheting upwards of the E_2_ allele (see Appendix S3, Figures S5–S9). It can be seen from further analyses that the fitness effects of extrapair interactions generally play a larger role than those of within‐pair interactions in promoting the evolution of E_2_ (see Appendix S3 Figure ,S8).

## 4 DISCUSSION

Increasing evidence provided by long‐term field data and/or meta‐analysis has indicated that female EPC behavior may not bring benefits to them, and therefore, nonadaptive models may be necessary to explain this behavior (Hsu, Schroeder, Winney, Burke, & Nakagawa, 2014; Hsu et al., 2015). However, such models have been neglected for quite a long time (Forstmeier et al., 2014). To our knowledge, this study is the first time that a nonadaptive hypothesis has been tested theoretically in this context.

Our “two‐locus hitchhiking” model is based on the hypotheses proposed by Sheldon (2000) and Forstmeier et al. (2011). For males, allocating more effort to seek EPCs would increase their extrapair fertility, but would simultaneously reduce the fitness of their social mates due to their reduced parental care investment. For females, however, completely accepting or pursuit behavior is not under direct selection in this model, but spreads because these females mate more often than randomly with males pursuing EPCs, causing a statistical association between the alleles for these behaviors. As shown in Figure 1, if there is no cost for E_2_ females (also no benefits), both behaviors can be maintained and remain polymorphic in the population, or even become fixed in the whole population. As the equilibrium points shown on the curve (Figure 1) are always neutrally stable, there will be no selection pressure causing the change of allele frequencies when the population achieves any of these points. However, perturbations arising from other factors like genetic drift, mutations, or pleiotropic effects may strongly affect the evolution of both female pursuit and male preference behavior, that is, displacing the population from one equilibrium point to another one on the line (see similar argument for the evolution of female mating preference in Kirkpatrick, 1982). Note that any cost from a decrease in male care to females that seek EPCs would prevent the evolution of female pursuit behavior, as we discuss more below.

Similar mechanisms have also been considered in other contexts, such as in providing a possible explanation for the evolution of polyandry. It was suggested that polyandry might evolve due to indirect selection, that is, genetic covariances with male fertilization efficiency (Keller & Reeve, 1995). This scenario, often known as the “sexually selected sperm” hypothesis, has also recently been developed through individual‐based models (Bocedi & Reid, 2015).

We also propose a second explanation for the evolution of nonadaptive female pursuit behavior. After adding another locus S representing the expression of sexual signaling traits in females into the two‐locus model, we find that the interlocus SAC between male allocation behavior (i.e., determined by the locus P) and female sexual signaling traits (i.e., determined by the locus S) is capable of producing both stable oscillating selection and stable oscillating linkage disequilibria between the E locus and the other two loci (Figure S2). The combination of these effects is consequently capable of producing stronger and longer‐term positive than negative indirect selection that increases the frequency of the allele E_2_ through a ratchet‐like mechanism (Figures S2 and S5–S7). In nature, it has long been realized that males can be choosy through the differential allocation of parental care (Sheldon, 2000), although research in this field is heavily biased toward considering differential allocation of parental care from a female perspective instead (Ratikainen & Kokko, 2010). In many species, breeding asynchrony enables males to gain extrapair matings even during the periods when males are providing parental care (Whittingham & Dunn, 2001), thereby establishing a trade‐off between providing parental care and seeking EPCs. Males are expected to arrange their time and effort in a way that maximizes their overall reproductive success (Westneat, Sherman, & Morton, 1990). This may lead to indirect selection that promotes the evolution of female pursuit behavior through the mechanism demonstrated above. Note that male‐seeking EPCs trading off against paternal care is a key structural assumption of the models presented in this study. There may be some other possible trade‐offs for male EPC behavior, such as pursuing EPCs versus defending within‐pair paternity (Akçay et al., 2012), which remains to be explored theoretically as drivers of the evolution of female EPC behavior in the future.

Pursuit behavior may sometimes be costly to females (Westneat & Stewart, 2003). We find that through linkage disequilibrium with male differential allocation behavior, pursuit behavior can evolve and be maintained only when there is no cost for females in our basic two‐locus hitchhiking model. Other mechanisms that can provide positive selection would be required to compensate for any costs. As shown in our “three‐locus male choice” model, the ratcheting effect that drives the evolution of female pursuit behavior is also somewhat sensitive to costs. Although the overall positive force of the indirect selection in this second model is relatively weak and may be easily overwhelmed by negative direct selection from the lowered male parental care (e.g., Figures S2 and S5), indirect selection can still play a role in promoting the evolution of female pursuit behavior, especially when this behavior is only mildly maladaptive to females. For example, if males have a quite low sensitivity to female pursuit behavior, for example, when they cannot easily detect female's pursuit behavior (Whittingham & Dunn, 2001) or would only occasionally reduce parental care after detecting it, female pursuit behavior can evolve gradually in this situation. Empirical tests of the costs of female EPC behavior are rare, and a conclusion on whether costs are generally expected remains ambiguous (Westneat & Stewart, 2003). Although reduction in male paternal care has been well‐cited, Whittingham and Dunn (2001) indicated that more than half of the studies they reviewed had no relationship between male parental care and paternity. Therefore, the indirect selection effects we explored in this study may be biologically important in nature. Those effects require more empirical and quantitative genetic evidence to verify and are still wide open for future studies. For example, the different trade‐offs for male EPC behavior discussed above may have relatively pervasive biological significance in understanding the evolution of female EPC behavior, which deserve further empirical studies to verify in the future. Such research will further promote our understanding of this interesting behavior of completely accepting or even pursuing EPCs in females.

## CONFLICT OF INTEREST

None declared.

## AUTHOR CONTRIBUTIONS

N.L. and Y.H.S. conceived the study. N.L. and M.R.S. performed the analyses. All authors wrote and edited the manuscript.
