# Supplementary material for: Nonadaptive female pursuit of extrapair copulations can evolve through hitchhiking
Source: Ecol Evol. 2018 Mar 6;8(7):3685–92. doi: 10.1002/ece3.3915 (PMC5901172; doi:10.1002/ece3.3915)
Supplement: Supplementary file 1 [file ECE3-8-3685-s001.docx]

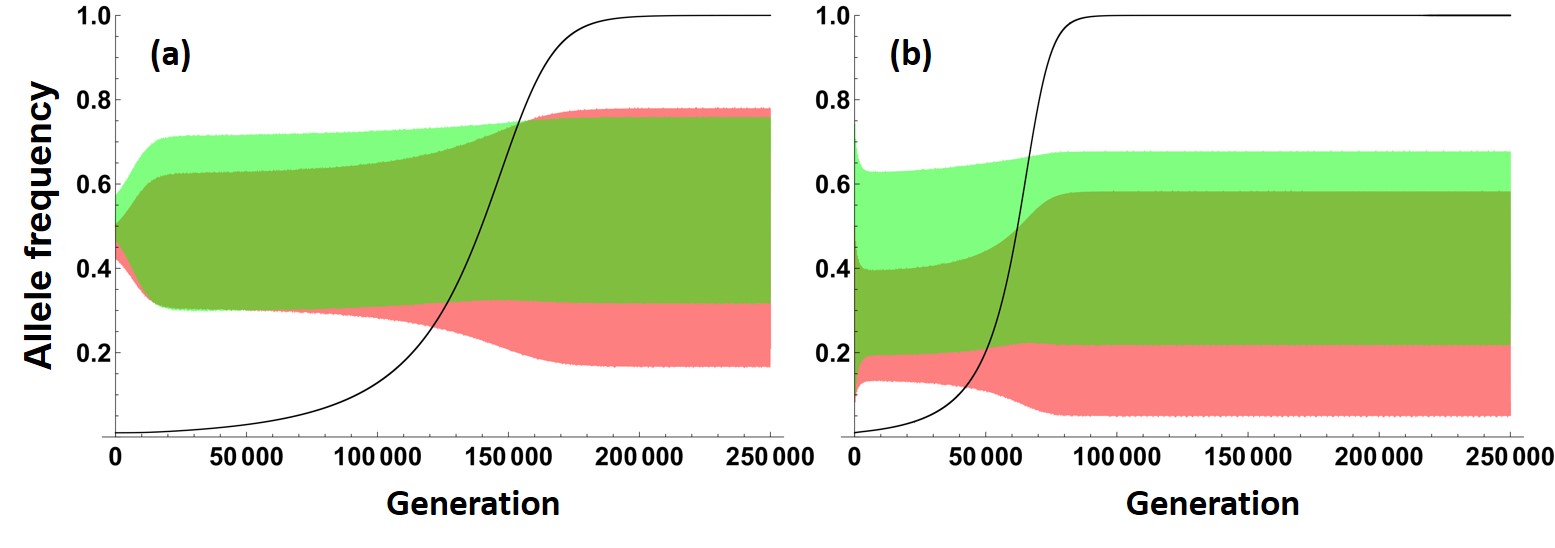


Figure S1. The dynamics of allele frequencies of p_2_ (red curve), s_2_ (green curve) and e_2_ (black curve) under the asymmetric scenario from Lyu et al. (2017). Similar to our previous stud (Lyu et al. 2017), here we assume that a P_2_ male will reduce his parental investment to $1 - \delta_{1}$ and increase his EPC effort to $c+d_{c1}$ when he mates with an S_1_ female, and increase his parental investment to $1+ \delta_{2}$ with $c-d_{c2}$ as his EPC effort when he mates with an S_2_ female, relative to that for a P_1_ male. The allele E_2_ also evolves in this situation due to the oscillating linkage disequilibrium, as shown in figure 2a. (a) $\delta_{1}=0.2$; $\delta_{2}=0.15$; $d_{c1}=0.38$; $d_{c2}=0.3$, (b) $\delta_{1}=0.3$; $\delta_{2}=0.35$; $d_{c1}=0.5$; $d_{c2}=0.6$. The other parameter values are: $b = 0.8$; $c = 0.8$; $t = 0.07$; $\mu=0.5$; $\tau=0.5$; $\delta^{'}=0.00001$.


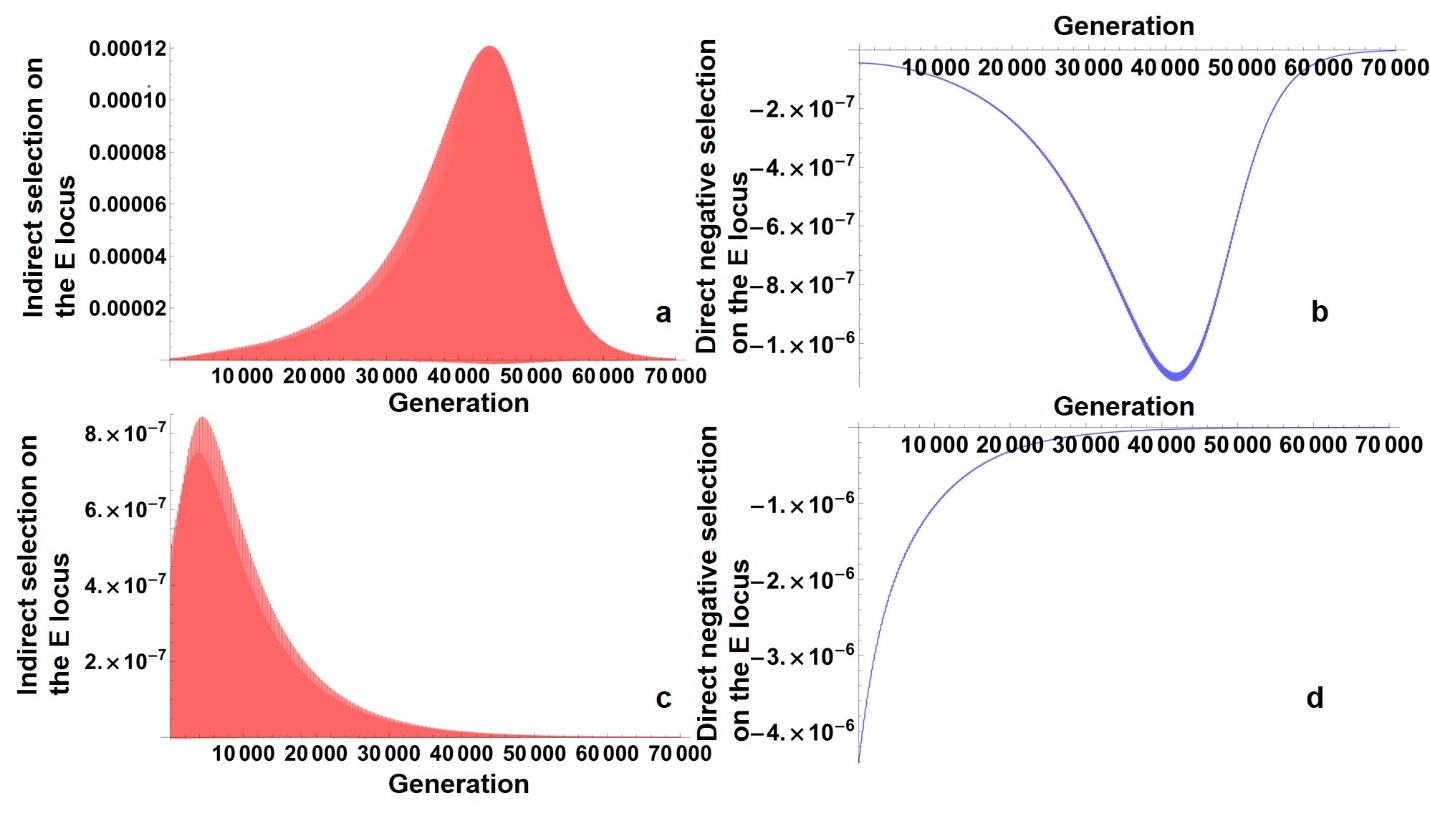


Figure S2. The indirect selection effects from linkage disequilibrium (a, c) and direct negative selection effect from the decreased male parental care (b, d) on the E_2_ allele. The frequency dynamics of allele E_2_ is determined by the direct negative selection from decreased male parental care, and the indirect selection brought by linkage disequilibria ($\Delta e_{2}={\Delta e}_{2}^{dir}+{\Delta e}_{2}^{ind}$, detailed see the equation S5 in the Appendix B). These curves consist of oscillations as shown in Figure S6 and S7. (a) and (b) correspond to Figure 2a, (c) and (d) correspond to figure 2b.


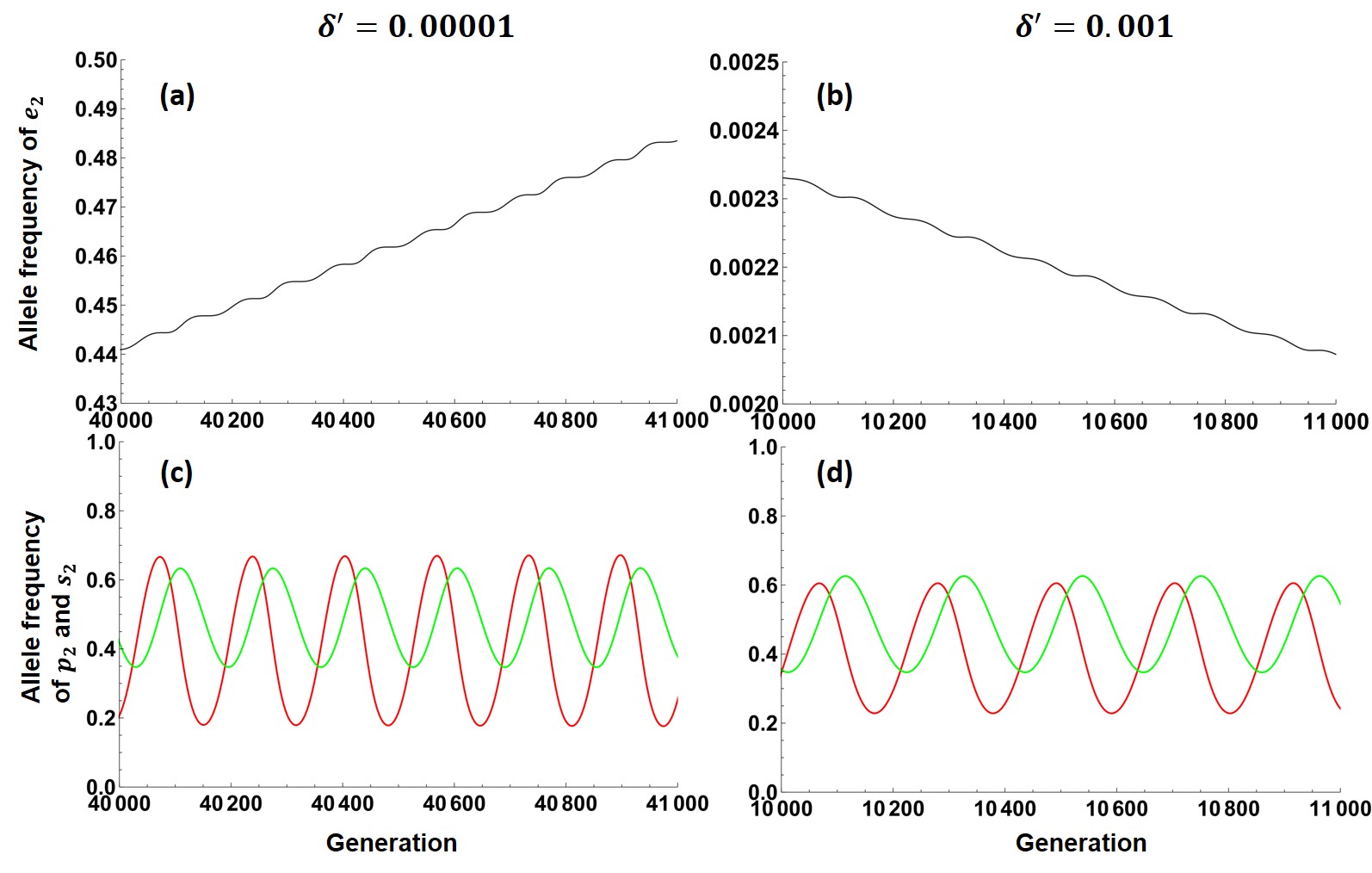


Figure S3. Magnified dynamics of allele frequencies of e_2_ (black curves in (a) and (b)), p_2_ (red curves in (c) and (d)), and s_2_ (green curves in (c) and (d)). Other parameters are the same as in Figure 2.


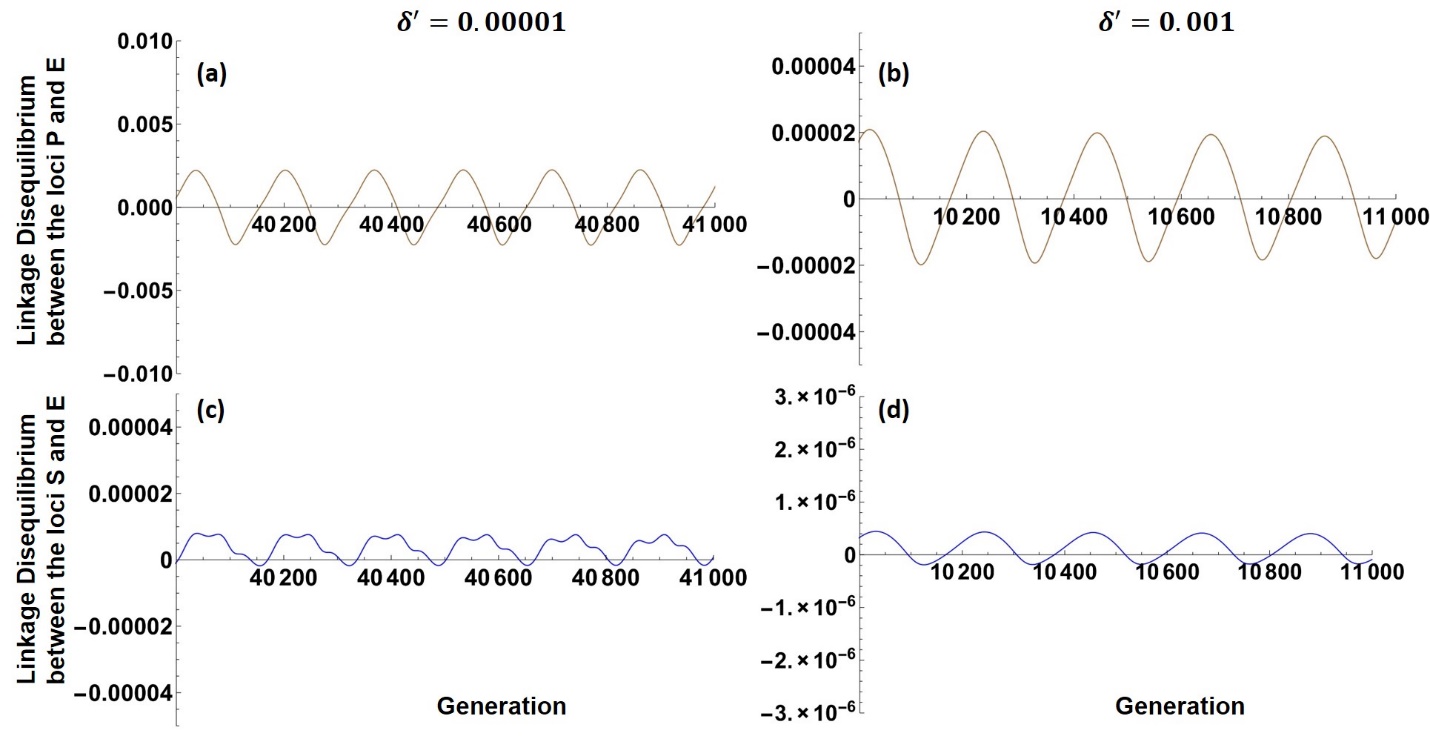


Figure S4. Magnified dynamics of linkage disequilibria D_PE_ (brown curves in (a) and (b)) and D_SE_ (blue curves in (c) and (d)). Other parameters are the same as in Figure 2.


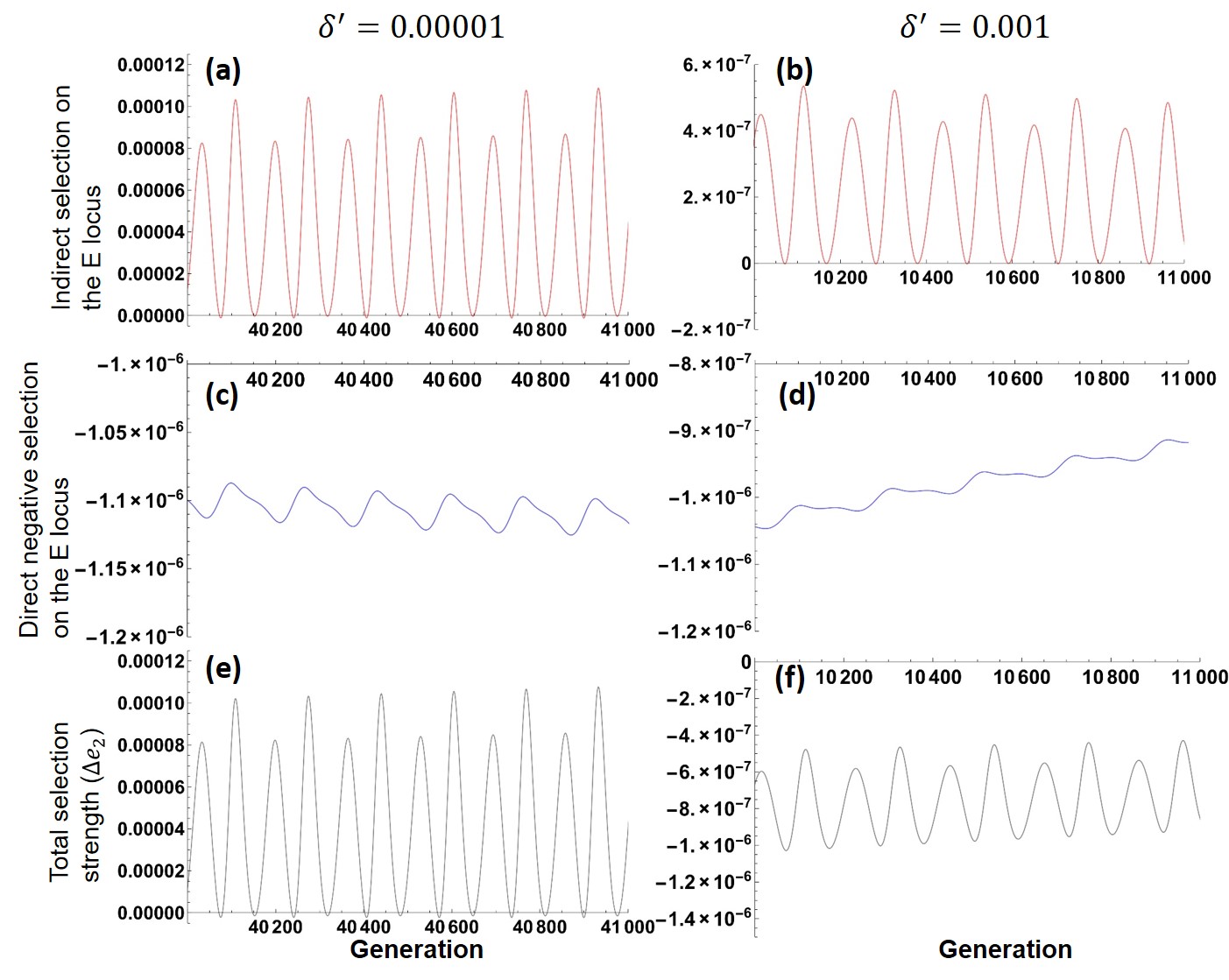


Figure S5. Magnified indirect selection effects from linkage disequilibrium (a, b), direct negative selection effect from the lowered male parental care (c, d), and the total selection strength on the allele E_2_ (e, f). The other parameter values are the same as in Figure 2.


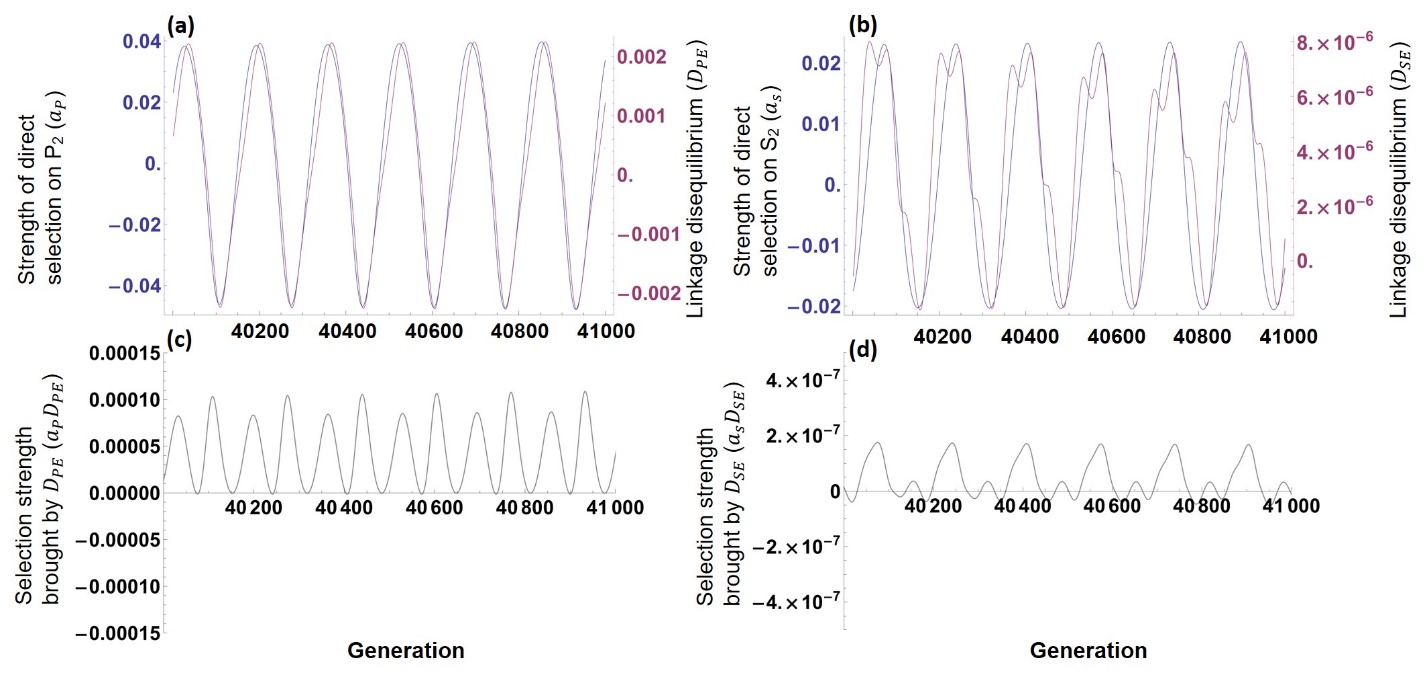


Figure S6. Magnified indirect selection effects from linkage disequilibrium $D_{PE}$ (a, c) and $D_{SE}$ (b, d) on the allele E_2_ when $\delta^{'}=0.00001$. The other parameter values are the same as in Figure 2.


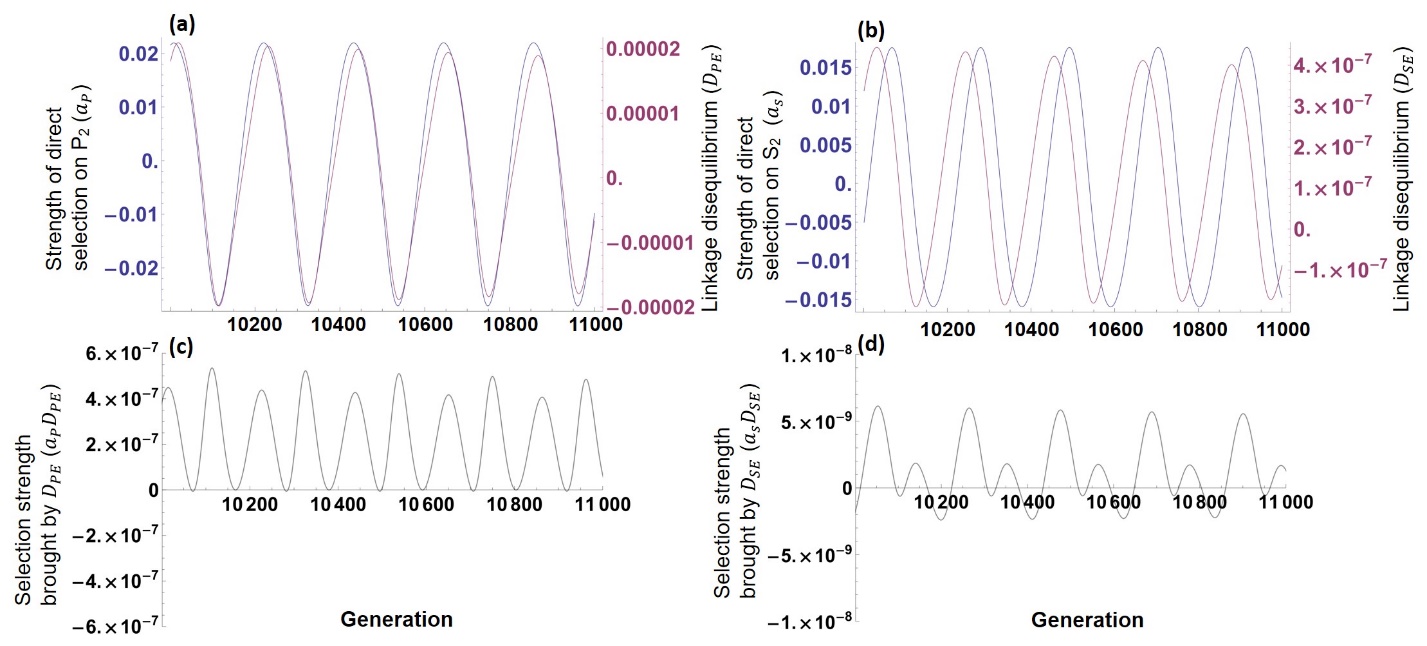


Figure S7. Magnified indirect selection effects from linkage disequilibrium $D_{PE}$ (a, c) and $D_{SE}$ (b, d) on the allele E_2_ when $\delta^{'}=0.001$. The other parameter values are the same as in Figure 2.


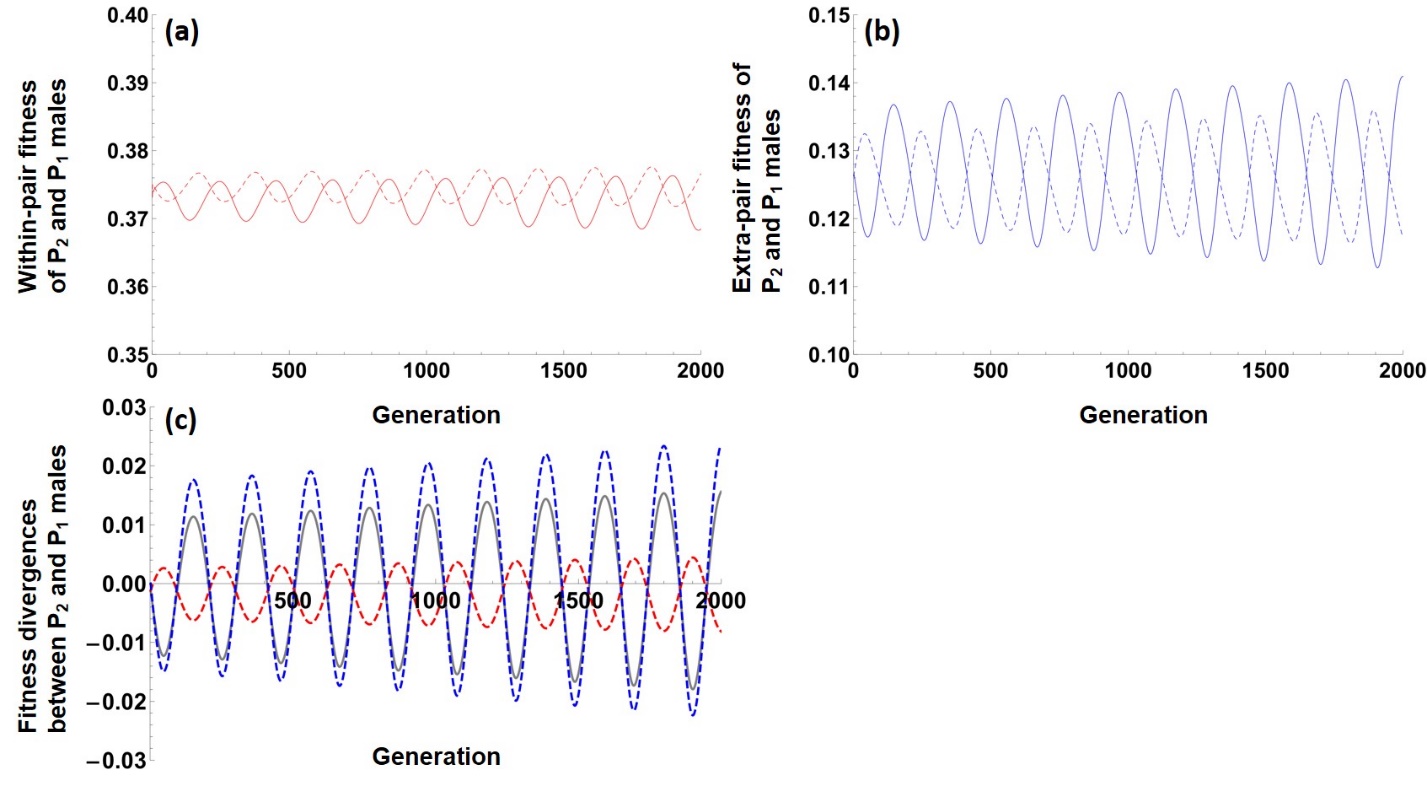


Figure S8. Within-pair and extra-pair fitness dynamics of P_2_ and P_1_ males and the fitness divergences between P_2_ and P_1_ males. In (a) and (b), the solid line represents the fitness of P_2_ males, and the dashed line represents the fitness of P_1_ males. (c) shows the fitness divergences, in which the red dashed line represents the within-pair divergence, the blue dashed line represents the extra-pair divergence, and the grey line represents the total divergence. The other parameter values are the same as in Figure 2a.


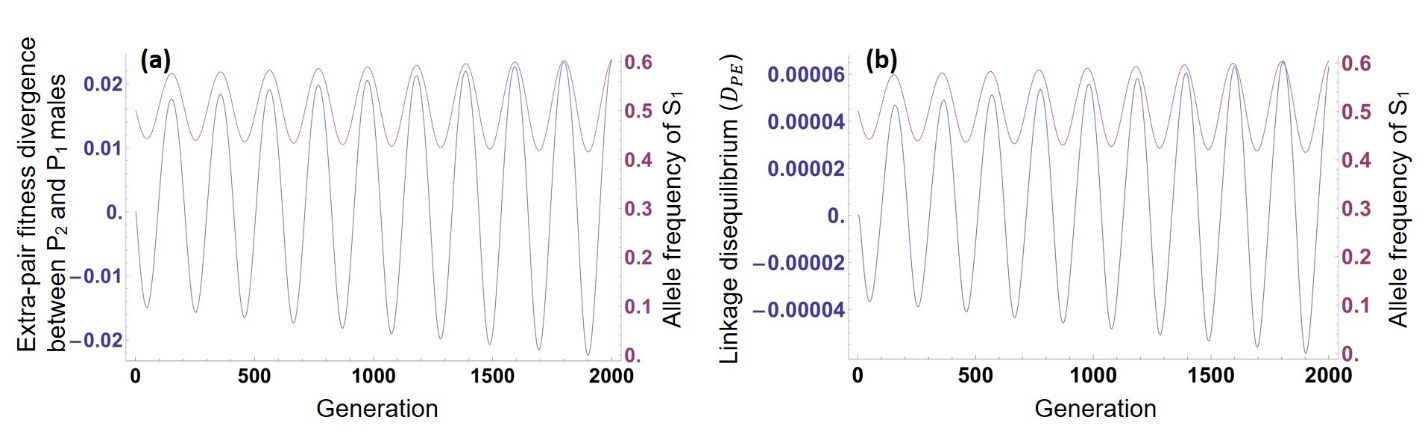


Figure S9. The synchronous dynamics. (a) shows the dynamics of the extra-pair fitness divergence between P_2_ and P_1_ males (blue curve) and the allele frequency of S_1_ (red curve), and (b) shows the dynamics of the linkage disequilibrium between the loci P and E (blue curve) and the allele frequency of S_1_ (red curve). The other parameter values are the same as in Figure 2a.
