# Supplementary material for: Nonadaptive female pursuit of extrapair copulations can evolve through hitchhiking
Source: Ecol Evol. 2018 Mar 6;8(7):3685–92. doi: 10.1002/ece3.3915 (PMC5901172; doi:10.1002/ece3.3915)
Supplement: Supplementary file 2 [file ECE3-8-3685-s002.docx]

Table S1. The surviving offspring matrix for each male-female mating combination in the “two-locus hitchhiking” model’. The frequencies of four genotypes P_1_E_1_, P_1_E_2_, P_2_E_1_ and P_2_E_2_ are $x_{1}, x_{2}, x_{3}\mathrm{and}x_{4}$, respectively. We can see that the surviving offspring $\emptyset_{11}=\emptyset_{13}=\emptyset_{21}=\emptyset_{23}$, we denote them as $\emptyset_{1}$ for describing the modeling outputs (see Appendix A). Similarly, we denote $\emptyset_{ij}$ as $\emptyset_{2}$ if$i=1 or 2$ and$j=2\mathrm{or}4$; as $\emptyset_{3}$ if$i=3 or 4$ and$j=1\mathrm{or}3$; as $\emptyset_{4}$ if$i=3 or 4$ and$j=2\mathrm{or}4$.

| Female genotype | Male genotype |  |
| --- | --- | --- |
|  | P_1_E_1_ or P_1_E_2_ | P_2_E_1_ or P_2_E_2_ |
| P_1_E_1_ or P_2_E_1_ | $\emptyset_{1}=1+b$ | $\emptyset_{3}=1+b(1-\delta)$ |
| P_1_E_2_ or P_2_E_2_ | $\emptyset_{2}=1+b(1-\delta^{'})$ | $\emptyset_{4}=1+b(1-\delta-\delta^{'})$ |

Table S2. The surviving offspring matrix for each male-female mating combination in the “three-locus male choice” model. The frequencies of eight genotypes P_1_S_1_E_1_, P_1_S_1_E_2_, P_1_S_2_E_1_, P_1_S_2_E_2_, P_2_S_1_E_1_, P_2_S_1_E_2_, P_2_S_2_E_1_ and P_2_S_2_E_2_ are $x_{1}, x_{2}, x_{3}\ldots x_{8}$, respectively. We can see that the surviving offspring $\emptyset_{11}=\emptyset_{15}=\emptyset_{21}=\emptyset_{25}=\emptyset_{31}=\emptyset_{35}=\emptyset_{41}=\emptyset_{45}$, we denote them as $\emptyset_{1}^{'}$ for describing the modeling outputs (see Appendix B and C). Similarly, we denote $\emptyset_{\mathrm{ij}}$ as $\emptyset_{2}^{'}$ if$i<5$ and$j=2\mathrm{or}6$; as $\emptyset_{3}^{'}$ if$i<5$ and$j=3\mathrm{or}7$; as $\emptyset_{4}^{'}$ if$i<5$ and$j=4\mathrm{or}8$; as $\emptyset_{5}^{'}$ if$i>4$ and$j=1 or 5$; as $\emptyset_{6}^{'}$ if$i>4$ and$j=2\mathrm{or}6$; as $\emptyset_{7}^{'}$ if$i>4$ and$j=3\mathrm{or}7$; as $\emptyset_{8}^{'}$ if$i>4$ and$j=4\mathrm{or}8$.

| Female genotype | Male genotype |  |  |
| --- | --- | --- | --- |
|  | P_1_S_1_E_1_, P_1_S_1_E_2_, P_1_S_2_E_1_ or P_1_S_2_E_2_ | P_2_S_1_E_1_, P_2_S_1_E_2_, P_2_S_2_E_1_ or P_2_S_2_E_2_ |  |
| P_1_S_1_E_1_ or P_2_S_1_E_1_ | $\emptyset_{1}^{'}=1+b$ | $\emptyset_{5}^{'}=1+b(1-\delta)$ |  |
| P_1_S_1_E_2_ or P_2_S_1_E_2_ | $\emptyset_{2}^{'}=1+b(1-\delta^{'})$ | $\emptyset_{6}^{'}=1+b(1-\delta-\delta^{'})$ |  |
| P_1_S_2_E_1_ or P_2_S_2_E_1_ | $\emptyset_{3}^{'}=(1+b)(1-t)$ | $\emptyset_{7}^{'}=[1+b\left( 1+\delta\right)](1-t)$ |  |
| P_1_S_2_E_2_ or P_2_S_2_E_2_ | $\emptyset_{4}^{'}=\left[ 1+b(1-\delta^{'}) \right](1-t)$ | $\emptyset_{8}^{'}=[1+b\left( 1+\delta-\delta^{'} \right)](1-t)$ |  |
