# Supplementary material for: Nonadaptive female pursuit of extrapair copulations can evolve through hitchhiking
Source: Ecol Evol. 2018 Mar 6;8(7):3685–92. doi: 10.1002/ece3.3915 (PMC5901172; doi:10.1002/ece3.3915)
Supplement: Supplementary file 3 [file ECE3-8-3685-s003.docx]

**Appendix A. “Two-locus hitchhiking” model outputs**

On one hand, if the female pursuit behavior is not costly (i.e., $\delta^{'}=0$), males will have the same fitness while mating with E_1_ or E_2_ females (i.e., $\emptyset_{1}=\emptyset_{2}$ and $\emptyset_{3}=\emptyset_{4}$, see Table S1). Therefore, we have

$\Delta p_{2}=\frac{1}{2}p_{2}\left[ \frac{\left( 1+b-b\delta\right)\left( 1-e_{1}\mu\tau-e_{2}\tau\right)+\sigma\left( c+d_{c} \right)}{w}-1 \right]$, (S1)

$\Delta e_{2}=\Delta p_{2}\frac{D}{p_{2}\left( 1-p_{2} \right)}$. (S2)

We can find that with $\delta^{'}=0$, the E_2_ allele will not be under direct selection, the frequency of this allele will therefore change only due to linkage disequilibrium, and$\Delta e_{2}$ will always be equal to zero when $D=0$ is satisfied (see in equation S2). We therefore can deduce that the lines on the edge of $p_{2}=0$ or $p_{2}=1$ are always equilibria. Local stabilities can be analyzed by examining the eigenvalues of the linearized dynamics around the equilibria. We find that the lines of equilibria are not always stable, or are stable only in part. Specifically, the line $p_{2}=0$ still requires $e_{2}<\frac{b\delta c\left( 1-\mu\tau\right)+d_{c}\left( 1+b \right)\mu\tau}{\left( d_{c}\left( 1+b \right)+b\delta c \right)\left( \tau-\mu\tau\right)}$ to enable it to be neutral stability, while the line $p_{2}=1$ requires $e_{2}>\frac{b\delta d_{c}+b\delta c\left( 1-\mu\tau\right)+d_{c}\left( 1+b \right)\mu\tau}{\left( d_{c}\left( 1+b \right)+b\delta c \right)\left( \tau-\mu\tau\right)}$. Furthermore, when $p_{2}=\left( \frac{1+b}{b\delta}+\frac{c}{d_{c}} \right)\left( \tau-\mu\tau\right)e_{2}-\frac{c}{d_{c}}\left( 1-\mu\tau\right)+\frac{1+b}{b\delta}\mu\tau$ is satisfied, both $\Delta p_{2}$ and $\Delta e_{2}$ will always be equal to zero. Numerical simulations suggest that this line is also neutrally stable (see Figure 1 in the main text).

On the other hand, if the pursuit of EPC behavior is costly to females (i.e., $\delta^{'}>0$), we have

$\Delta p_{2}^{'}=\frac{1}{2}p_{2}\left[ \frac{\left( 1+b-b\delta\right)\left( 1-e_{1}\mu\tau-e_{2}\tau\right)-b\delta^{'}e_{2}\left( 1-\tau\right)+\sigma\left( c+d_{c} \right)}{w}-1 \right]-\frac{b\delta^{'}D}{2w}$, (S3)

$\Delta e_{2}^{'}=\frac{1}{2}D\left[ \frac{\sigma d_{c}-b\delta\left( 1-e_{1}\mu\tau-e_{2}\tau\right)}{w} \right]-\frac{e_{1}e_{2}b\delta^{'}}{2w}$. (S4)

We can then generate the equilibria of the model on the edges or corners, which are given below in the form of $(\hat{p}$, $\hat{e}$, $\hat{D})$. They are $\left( \frac{1+b}{b\delta}\mu\tau-\frac{c}{d_{c}}\left( 1-\mu\tau\right), 0, 0 \right)$, $\left( \frac{1+b}{b\delta}\tau-\frac{c}{d_{c}}\left( 1-\tau\right)-\frac{\delta^{'}}{\delta}\tau, 1, 0 \right)$ and the four corners of (0, 0, 0), (0, 1, 0), (1, 0, 0) and (1, 1, 0). Stability analyses reveal that among those four cases of two-locus fixation, only (0, 0, 0) and (1, 0, 0) can be locally stable when $\frac{d_{c}}{c}<\frac{b\delta(1-\mu\tau)}{\left( 1+b \right)\mu\tau}$, and $\frac{d_{c}}{c}>\frac{b\delta(1-\mu\tau)}{\left( 1+b \right)\mu\tau-b\delta}$ are satisfied, respectively. Note that the two equilibria on the edges exist only when $\frac{b\delta(1-\mu\tau)}{\left( 1+b \right)\mu\tau}<\frac{d_{c}}{c}<\frac{b\delta(1-\mu\tau)}{\left( 1+b \right)\mu\tau-b\delta}$ and $\frac{b\delta\left( 1-\tau\right)}{\left( 1+b-b\delta^{'} \right)\tau}<\frac{c}{d_{c}}<\frac{b\delta\left( 1-\tau\right)}{\left( 1+b-b\delta^{'} \right)\tau-b\delta}$ are satisfied, respectively. Stability analyses further reveal that only the first equilibrium $\left( \frac{1+b}{b\delta}\mu\tau-\frac{c}{d_{c}}\left( 1-\mu\tau\right), 0,0 \right)$ can be stable while it exists (i.e., $\frac{b\delta(1-\mu\tau)}{\left( 1+b \right)\mu\tau}<\frac{d_{c}}{c}<\frac{b\delta(1-\mu\tau)}{\left( 1+b \right)\mu\tau-b\delta}$ ). We also concluded from numerical simulations that this model will have no stable internal equilibrium. Parameter ranges investigated included the relative effect of male, compared to female, parental care on offspring survival *b* from the set {0.5, 0.7, 0.9}, changes in parental care (i.e., $\delta$ and $\delta^{'}$) from the set {0.1, 0.2, 0.3, 0.4, 0.5}, and the proportion of extra-pair offspring produced by females while engaging in EPCs (i.e., *𝜏*), the probability of engaging in EPCs for E_1_ females (i.e., $\mu$) and the change in potential extra-pair benefits (i.e., $d_{c}$) from the set {0.2, 0.4, 0.6, 0.8, 1.0}.

**Appendix B. Direct and indirect selection on the allele E_2_ in the “three-locus male choice” model**

We derive the expression of the recursion equation ($\Delta e_{2}$) using the notation of Barton and Turelli (1991) (calculations are available in the *Mathematica* files on Dryad (to be submitted upon acceptance)), which can be written as

$\Delta e_{2}=-\frac{e_{1}e_{2}b\delta^{'}}{2w}\left( 1-s_{2}t \right)+a_{P}D_{PE}+a_{s}D_{SE}$, (S5)

where $a_{P}=\frac{1}{2w}\left( w_{a}-w_{b} \right)$, $a_{s}=\frac{1}{2w}\left( w_{c}-w_{d}-e_{2}b\delta^{'}t \right)$, where $w_{a}=x_{S_{1}E_{1}}\emptyset_{5}^{'}\left( 1-\mu\tau\right)+x_{S_{1}E_{2}}\emptyset_{6}^{'}\left( 1-\tau\right)+x_{S_{2}E_{1}}\emptyset_{7}^{'}\left( 1-\mu\tau\right)+x_{S_{2}E_{2}}\emptyset_{8}^{'}\left( 1-\tau\right)+\sigma^{'}\left( s_{1}\left( c+d_{c} \right)+s_{2}\left( c-d_{c} \right) \right)$, $w_{b}=x_{S_{1}E_{1}}\emptyset_{1}^{'}\left( 1-\mu\tau\right)+x_{S_{1}E_{2}}\emptyset_{2}^{'}\left( 1-\tau\right)+x_{S_{2}E_{1}}\emptyset_{3}^{'}\left( 1-\mu\tau\right)+x_{S_{2}E_{2}}\emptyset_{4}^{'}\left( 1-\tau\right)+\sigma^{'}c$, $w_{c}=p_{1}\emptyset_{4}^{'}+p_{2}\emptyset_{8}^{'}$ and $w_{d}=p_{1}\emptyset_{2}^{'}+p_{2}\emptyset_{6}^{'}$, where $\sigma^{'}=\frac{\sum_{i,j} x_{i}x_{j}\emptyset_{ij}^{'}\theta_{i}}{\sum_{i,j} x_{i}x_{j}C_{ij}}$, $\emptyset_{1}^{'}$, …$\emptyset_{8}^{'}$ can be found in Table S2, $x_{S_{1}E_{1}}$, $x_{S_{1}E_{2}}$, $x_{S_{2}E_{1}}$, and $x_{S_{2}E_{2}}$ represent the frequency of $S_{1}E_{1}$, $S_{1}E_{2}$,$S_{2}E_{1}$ and $S_{2}E_{2}$ respectively, and $D_{PE}$ and $D_{SE}$ represent the linkage disequilibria between the locus P and the locus E, and between the locus S and the locus E.

**Appendix C. Evolutionary mechanism of female pursuit behavior under the cycling behavior of P and S**

As the indirect selection (i.e., $a_{P}$) cycles between negative and positive values, so does the linkage disequilibrium between the loci P and E (Figure S6a, S7a). More importantly, they cycle nearly synchronously, which enables the corresponding selection strength on the allele E_2_ to be kept positive in the large majority of the time (Figure S6c, S7c). The linkage disequilibrium between the loci S and E shows a similar effect on the allele E_2_, but with much a smaller strength than that of the dynamics from the linkage disequilibrium between the locus P and E (Figure S6d, S7d).

Furthermore, we find that the selection strength $a_{P}$ can be treated as the fitness divergence between the P_2_ (i.e., $w_{a}$) and P_1_ males (i.e., $w_{b}$) according to our derivation of the recursion equation (equation S5). This equation can be divided into the fitness divergences emerging from within-pair (see the first four terms of $w_{a}$ and of $w_{b}$ from equation S5, i.e., $x_{S_{1}E_{1}}\emptyset_{5}^{'}\left( 1-\mu\tau\right)+x_{S_{1}E_{2}}\emptyset_{6}^{'}\left( 1-\tau\right)+x_{S_{2}E_{1}}\emptyset_{7}^{'}\left( 1-\mu\tau\right)+x_{S_{2}E_{2}}\emptyset_{8}^{'}\left( 1-\tau\right)$ and $x_{S_{1}E_{1}}\emptyset_{1}^{'}\left( 1-\mu\tau\right)+x_{S_{1}E_{2}}\emptyset_{2}^{'}\left( 1-\tau\right)+x_{S_{2}E_{1}}\emptyset_{3}^{'}\left( 1-\mu\tau\right)+x_{S_{2}E_{2}}\emptyset_{4}^{'}\left( 1-\tau\right)$) respectively, and extra-pair interactions (see the last remaining term of $w_{a}$ and $w_{b}$ of equation S5, i.e., $\sigma^{'}\left( s_{1}\left( c+d_{c} \right)+s_{2}\left( c-d_{c} \right) \right)$ and $\sigma^{'}c$ between the P_2_ and P_1_ males. We find that although the within-pair fitness is higher than the extra-pair fitness (Figure S8a, b), the extra-pair fitness divergence between the P_2_ and P_1_ males is generally higher than the within-pair divergence when the cycling happens (Figure S8c). Therefore, the extra-pair fitness effects should play a leading role in promoting the evolution of the allele E_2_. We can see that the three-locus male choice model in this study reduces to our previous two-locus model (with the loci P and S, Lyu et al. (2017)), when all females produce the same ratio of extra-pair offspring (e.g., when the allele frequency of E_2_ is equal to zero). As indicated in that study (Lyu et al. 2017), the benefit or cost from EPCs by P_2_ males (mating with S_1_ or S_2_ females, respectively) are always larger than the within-pair fitness cost or benefit when cycling happens. This is quite consistent with our findings in this study (Figure S8).

Finally, we find that the dynamics of both the difference of the fitness of P_2_ and P_1_ males through extra-pair effects and the linkage disequilibrium $D_{PE}$ are quite synchronous with the frequency dynamics of the allele S_1_. Intuitively, if the frequency of allele S_1_ increases in the population, there will be more P_2_ males allocating increased efforts to seek EPCs after mating with S_1_ females, which will subsequently intensify the extra-pair fitness divergence between the P_2_ and P_1_ males (i.e., increasing the selection strength $a_{P}$). Additionally, the P_2_ males will simultaneously have an increased probability of having extra-pair offspring with E_2_ females in this situation, which results in an increase of the linkage disequilibrium between the loci P and E. In contrast, if the allele frequency of S_1_ decreases, the extra-pair fitness divergence between the P_2_ and P_1_ males will decline, and even switch sign from positive to negative (Figure S9). In this situation, the P_2_ males would rather pay more attention to parental care (after mating with S_2_ females), and P_1_ males would have relatively high extra-pair fitness in the population. Likewise, P_1_ males will have an increased probability of having extra-pair offspring with E_2_ females in this situation, which results in lowered (or even negative) linkage disequilibrium between the loci P and E (Figure S9).

**References**

Barton, N. and M. Turelli. 1991. Natural and sexual selection on many loci. Genetics 127:229-255.

Lyu, N., M. R. Servedio, H. Lloyd, and Y. H. Sun. 2017. The evolution of postpairing male mate choice. Evolution 71:1465-1477.
